# Genetic Complexities of Cerebral Small Vessel Disease, Blood Pressure, and Dementia

**DOI:** 10.1001/jamanetworkopen.2024.12824

**Published:** 2024-05-22

**Authors:** Muralidharan Sargurupremraj, Aicha Soumaré, Joshua C. Bis, Ida Surakka, Tuuli Jürgenson, Pierre Joly, Maria J. Knol, Ruiqi Wang, Qiong Yang, Claudia L. Satizabal, Alexander Gudjonsson, Aniket Mishra, Vincent Bouteloup, Chia-Ling Phuah, Cornelia M. van Duijn, Carlos Cruchaga, Carole Dufouil, Geneviève Chêne, Oscar L. Lopez, Bruce M. Psaty, Christophe Tzourio, Philippe Amouyel, Hieab H. Adams, Hélène Jacqmin-Gadda, Mohammad Arfan Ikram, Vilmundur Gudnason, Lili Milani, Bendik S. Winsvold, Kristian Hveem, Paul M. Matthews, W. T. Longstreth, Sudha Seshadri, Lenore J. Launer, Stéphanie Debette

**Affiliations:** 1 Bordeaux Population Health Research Center, University of Bordeaux, Inserm, UMR 1219, Bordeaux, France; 2 Glenn Biggs Institute for Alzheimer’s & Neurodegenerative Diseases, University of Texas Health Sciences Center, San Antonio; 3 Cardiovascular Health Research Unit, Department of Medicine, University of Washington, Seattle; 4 Department of Internal Medicine, University of Michigan, Ann Arbor; 5 Estonian Genome Centre, Institute of Genomics, University of Tartu, Tartu, Estonia; 6 Department of Epidemiology, Erasmus MC University Medical Center, Rotterdam, the Netherlands; 7 School of Public Health, Boston University and the National Heart, Lung, and Blood Institute Framingham Heart Study, Boston, Massachusetts; 8 Department of Neurology, Boston University School of Medicine, Boston, Massachusetts; 9 Icelandic Heart Association, Kopavogur, Iceland; 10 Department of Neurology, Washington University School of Medicine & Barnes-Jewish Hospital, St Louis, Missouri; 11 NeuroGenomics and Informatics Center, Washington University in St Louis, St Louis, Missouri; 12 Nuffield Department of Population Health, University of Oxford, Oxford, United Kingdom; 13 Department of Psychiatry, Washington University School of Medicine, St Louis, Missouri; 14 Charles F. and Joanne Knight Alzheimer Disease Research Center, Washington University School of Medicine, St Louis, Missouri; 15 Department of Public Health, CHU de Bordeaux, Bordeaux, France; 16 Department of Neurology, University of Pittsburgh School of Medicine, Pittsburgh, Pennsylvania; 17 Department of Psychiatry, University of Pittsburgh School of Medicine, Pittsburgh, Pennsylvania; 18 Department of Epidemiology, University of Washington, Seattle; 19 Department of Health Systems and Population Health, University of Washington, Seattle; 20 INSERM U1167, University of Lille, Institut Pasteur de Lille, Lille, France; 21 Department of Epidemiology and Public Health, CHRU de Lille, Lille, France; 22 Department of Human Genetics, Radboud University Medical Center, Nijmegen, the Netherlands; 23 Latin American Brain Health (BrainLat), Universidad Adolfo Ibáñez, Santiago, Chile; 24 Faculty of Medicine, University of Iceland, Reykjavik, Iceland; 25 Division of Clinical Neuroscience, Department of Research and Innovation, Oslo University Hospital, Oslo, Norway; 26 K. G. Jebsen Center for Genetic Epidemiology, Department of Public Health and Nursing, Faculty of Medicine and Health Sciences, Norwegian University of Science and Technology (NTNU), Trondheim, Norway; 27 Department of Neurology, Oslo University Hospital, Oslo, Norway; 28 HUNT Research Centre, Department of Public Health and Nursing, Norwegian University of Science and Technology, Levanger, Norway; 29 Department of Brain Sciences, Imperial College London, London, United Kingdom; 30 UK Dementia Research Institute, Imperial College London, London, United Kingdom; 31 Data Science Institute, Imperial College London, London, United Kingdom; 32 Department of Neurology, University of Washington, Seattle; 33 Laboratory of Epidemiology and Population Sciences, Intramural Research Program, National Institute on Aging, Bethesda, Maryland; 34 Institute for Neurodegenerative Diseases, Department of Neurology, Bordeaux University Hospital, Bordeaux, France

## Abstract

**Importance:**

Vascular disease is a treatable contributor to dementia risk, but the role of specific markers remains unclear, making prevention strategies uncertain.

**Objective:**

To investigate the causal association between white matter hyperintensity (WMH) burden, clinical stroke, blood pressure (BP), and dementia risk, while accounting for potential epidemiologic biases.

**Design, Setting, and Participants:**

This study first examined the association of genetically determined WMH burden, stroke, and BP levels with Alzheimer disease (AD) in a 2-sample mendelian randomization (2SMR) framework. Second, using population-based studies (1979-2018) with prospective dementia surveillance, the genetic association of WMH, stroke, and BP with incident all-cause dementia was examined. Data analysis was performed from July 26, 2020, through July 24, 2022.

**Exposures:**

Genetically determined WMH burden and BP levels, as well as genetic liability to stroke derived from genome-wide association studies (GWASs) in European ancestry populations.

**Main Outcomes and Measures:**

The association of genetic instruments for WMH, stroke, and BP with dementia was studied using GWASs of AD (defined clinically and additionally meta-analyzed including both clinically diagnosed AD and AD defined based on parental history [AD-meta]) for 2SMR and incident all-cause dementia for longitudinal analyses.

**Results:**

In 2SMR (summary statistics–based) analyses using AD GWASs with up to 75 024 AD cases (mean [SD] age at AD onset, 75.5 [4.4] years; 56.9% women), larger WMH burden showed evidence for a causal association with increased risk of AD (odds ratio [OR], 1.43; 95% CI, 1.10-1.86; *P* = .007, per unit increase in WMH risk alleles) and AD-meta (OR, 1.19; 95% CI, 1.06-1.34; *P* = .008), after accounting for pulse pressure for the former. Blood pressure traits showed evidence for a protective association with AD, with evidence for confounding by shared genetic instruments. In the longitudinal (individual-level data) analyses involving 10 699 incident all-cause dementia cases (mean [SD] age at dementia diagnosis, 74.4 [9.1] years; 55.4% women), no significant association was observed between larger WMH burden and incident all-cause dementia (hazard ratio [HR], 1.02; 95% CI, 1.00-1.04; *P* = .07). Although all exposures were associated with mortality, with the strongest association observed for systolic BP (HR, 1.04; 95% CI, 1.03-1.06; *P* = 1.9 × 10^−14^), there was no evidence for selective survival bias during follow-up using illness-death models. In secondary analyses using polygenic scores, the association of genetic liability to stroke, but not genetically determined WMH, with dementia outcomes was attenuated after adjusting for interim stroke.

**Conclusions:**

These findings suggest that WMH is a primary vascular factor associated with dementia risk, emphasizing its significance in preventive strategies for dementia. Future studies are warranted to examine whether this finding can be generalized to non-European populations.

## Key Points

**Question:**

Do genetic instrumental variable analyses provide evidence of causation between vascular traits and Alzheimer disease (AD)?

**Findings:**

Using mendelian randomization (MR), this study showed a putative causal association of larger white matter hyperintensity (WMH) burden with increased AD risk after accounting for pulse pressure effects, and association of lower BP with AD risk with possible confounding by shared genetic instruments. Longitudinal analyses on individual-level data supported the association of genetically determined larger WMH with incident all-cause dementia and AD, independently of interim stroke.

**Meaning:**

With the use of complementary genetic epidemiology approaches, these findings suggest that WMH is a primary vascular factor associated with dementia risk.

## Teaser

This study investigates associations of genetically defined vascular traits with all-cause dementia and Alzheimer disease, while accounting for potential epidemiologic biases.

## Introduction

With increasing life expectancy, the prevalence of dementia is expected to reach 75 million by 2030.^1,2^ Devising strategies to prevent or delay its occurrence is a major public health priority. It is now widely recognized by the scientific community that most dementia cases in the population, including Alzheimer disease (AD), are related to a combination of vascular and neurodegenerative lesions.^3,4,5,6^ On postmortem examinations, 80% of patients with clinically diagnosed AD have cerebrovascular lesions.^7^ Among patients with stroke, the risk of incident dementia is at least doubled.^8,9^ At the population level, covert cerebral small vessel disease, detectable on brain imaging in the absence of clinical stroke, is thought to be the main pathologic substrate underlying the vascular contribution to cognitive decline and dementia,^10^ with nearly half of dementia cases exhibiting both AD and cerebral small vessel disease neuropathologic characteristics.^11^

White matter hyperintensity (WMH) burden is the most common cerebral small vessel disease feature on brain magnetic resonance imaging. Evidence from observational studies has established strong associations of WMH with increased risk of stroke and dementia, including AD,^12^ yet evidence for causality is limited. A putative causal association has been suggested in a preliminary mendelian randomization (MR) analysis that used genetic instruments as proxies for WMH volume, thus leveraging the natural randomization of genetic variation at conception to mitigate risks of confounding and reverse causation inherent to observational studies.^13,14^ However, while high blood pressure (BP) is by far the strongest risk factor for WMH, with extensive shared genetic variation,^13^ several MR studies have reported inverse associations of genetically determined BP levels^15^ with AD. These associations were observed both in datasets using standard AD diagnostic criteria^16,17,18^ and in studies additionally using self-reported parental history as a proxy for AD diagnosis.^19^ Complex age-dependent effects, possibly associated with the disease process, may lead to methodological issues, such as selective survival,^20^ and intrinsic structural changes, such as arterial stiffness^21^ and neurodegenerative lesions in BP-regulated regions, resulting in reverse causation.^22,23^ However, these inconsistencies remain poorly understood. A better understanding of the causal associations of vascular traits with AD risk is crucial to prioritize interventions and optimally target populations to prevent cognitive decline and dementia. Here, taking a multipronged genetic epidemiologic approach, we aim to systematically examine putative causal associations of genetically defined vascular traits with all-cause dementia and AD, while ruling out potential biases.

## Methods

**Figure 1.**
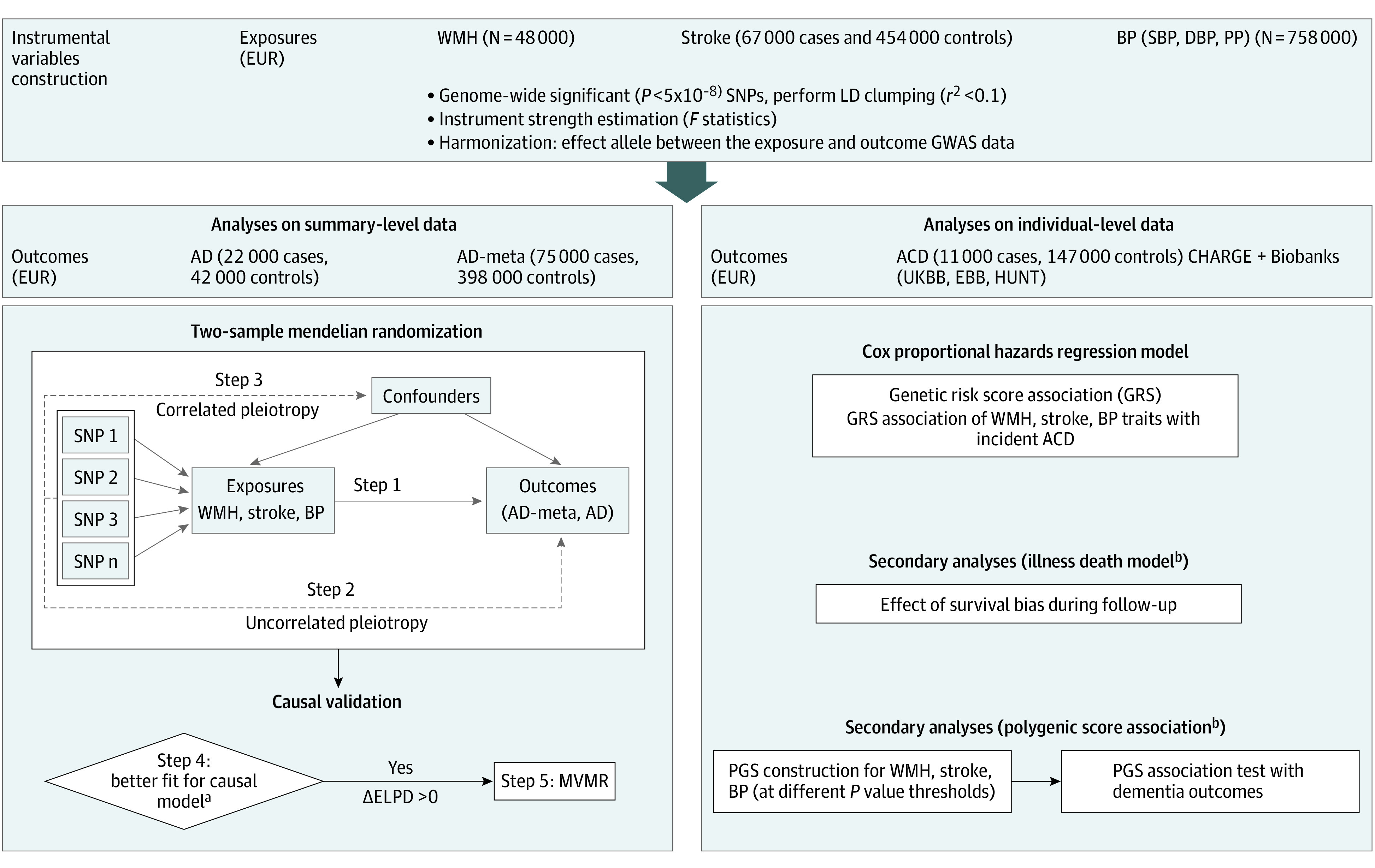
Study Design Analyses on summary-level data: In step 1, we used the standard inverse variance weighting method to estimate causal effects between each exposure and Alzheimer disease (AD) or AD-meta with parental history of dementia. Steps 2 and 3 addressed potential pleiotropic effects confounding the initial causal estimates using MR-RAPS, weighted-median and mode-based methods. ACD indicates all-cause dementia; BP, blood pressure; CHARGE, Cohorts for Heart and Aging Research in Genomic Epidemiology; DBP, diastolic blood pressure; EBB, Estonian Biobank; EUR, European population; GWAS, genome-wide association study; HUNT, Trøndelag Health Study; LD, linkage disequilibrium; PGS, polygenic profile score; PP, pulse pressure; SBP, systolic blood pressure; SNP, single-nucleotide polymorphism; UKBB, UK Biobank; and WMH, white matter hyperintensity. ^a^In step 4, we compared the causal model with the sharing model using MR-CAUSE. The risk factor–outcome associations favoring the causal model (change in expected log pointwise posterior density [ΔELPD] >0; see Methods) were validated in step 5 using multivariable mendelian randomization (MVMR). ^b^Association analyses in a subset of CHARGE cohorts (Three-City study, Ages Gene/Environment Susceptibility study).

We used 2 complementary approaches to examine the association of vascular traits (WMH, stroke, and BP) with dementia risk (Figure 1). First, we used summary-level data from published genome-wide association study (GWAS) meta-analyses to examine putative causal associations in a 2-sample MR (2SMR) framework. These GWASs were based on cross-sectional studies with mostly clinic-based (stroke, dementia) or population-based (WMH, BP) recruitment.^13,15,24,25,26^ Second, we leveraged individual-level data from 13 longitudinal cohorts and biobanks with prospective dementia surveillance to examine the association of weighted genetic risk scores (wGRSs) for WMH, stroke, and BP with incident dementia using Cox proportional hazards regression models. Secondary analyses were conducted in 2 cohorts with participants aged 65 years or older (the Ages Gene/Environment Susceptibility [AGES] study^27^ and the Three-City [3C] study^28^) using multistate models accounting for selective survival bias and polygenic scores. The MR study adhered to the Strengthening the Reporting of Observational Studies in Epidemiology (STROBE) reporting guideline,^29^ and the genetic association analyses followed the Strengthening the Reporting of Genetic Association Studies (STREGA) reporting guideline.^30^ Cohorts included in individual-level analyses were approved by the relevant ethics committees and institutional review boards (eTable 3 in Supplement 1).

### Analyses on Summary-Level Data

Two-sample MR uses single-nucleotide polymorphisms as genetic instruments for a given exposure (WMH, stroke, BP traits) to assess their putative causal association with the outcome (dementia). The validity of causal estimates relies on the assumption that these instruments are (1) strongly associated with the exposure (relevance) and (2) independent of the outcome given the exposure and confounders (independence) and (3) that the causal association is exclusively mediated by the exposure (exclusion restriction).

#### Exposures

Genetic instruments for exposures were derived from European ancestry GWASs based on 48 454 population-based participants for WMH, 67 162 cases and 454 450 controls for stroke, and 757 601 population-based participants for systolic BP (SBP), diastolic BP (DBP), and pulse pressure (PP), of which the study design was described previously.^13,15,24^ Cases in stroke GWASs were derived from both clinic-based and population-based studies and comprised patients with any stroke (ischemic stroke, intracerebral hemorrhage, or stroke of unknown or undetermined type), while controls were free of any stroke. In the BP GWASs, 15 mm Hg was added to SBP and 10 mm Hg was added to DBP for individuals taking BP-lowering medication. For each exposure, only independent genome-wide significant single-nucleotide polymorphisms (*P* < 5 × 10^−8^; *r*^2^ < 0.1) were considered. Instrument strength was assessed using the Cragg-Donald *F* statistic to meet the relevance MR assumption (eMethods in Supplement 2).^31,32^

#### Outcomes

For dementia outcomes, we used European association statistics from GWASs of clinically diagnosed late-onset AD (21 982 cases and 41 944 controls)^25^ and additionally meta-analyzed including both clinically diagnosed AD and broadly defined AD using self-reported parental history as a proxy for AD diagnosis (hereafter, *AD-meta*) that included both clinical AD cases (n = 21 982) and AD-meta cases based on parental history of dementia (n = 53 042) from the UK Biobank.^26^ The AD-meta phenotype is a pseudolinear measure of AD risk incorporating the participant’s dementia diagnosis weighted on parental dementia diagnoses and age, which was shown to have a near-unit correlation with clinical diagnosis.^33,34^

#### Causal Effect Estimation

In step 1, the inverse variance weighting method was used to estimate the putative causal association of WMH, stroke, SBP, DBP, and PP with AD (Figure 1). Step 2 aimed at testing the exclusion restriction MR assumption, using a suite of pleiotropy-robust methods (MR-RAPS, weighted median and mode) to account for potential effects of genetic instruments directly on the outcome that are uncorrelated with the exposure (uncorrelated pleiotropy).^35^ Step 3 aimed at testing the independence MR assumption, using a bayesian approach that addresses correlated pleiotropy (MR-CAUSE), ensuring the independence of instruments from both exposure and outcome through confounders (Figure 1; eMethods in Supplement 2).^36^ Two-sample MR analyses rely on published GWASs that are mostly adjusted for age and sex but not for other potential confounders. MR-CAUSE enables estimation of causal effects accounting for “unmeasured” confounding. When all instruments exhibit correlation for their effects on exposure and outcome, MR-CAUSE favors a causal model (γ) over the sharing model (*q*) in which pleiotropy due to confounders results in correlation only for a subset of instruments.^36^ A positive difference in expected log pointwise posterior density (ΔELPD = ELPDγ − ELPD*q*) indicates the causal model’s superiority (eMethods in Supplement 2). In step 4, for exposure-outcome pairs in which MR-CAUSE indicated a better fit for the causal model (ΔELPD > 0) but evidence for a significant sharing model (*P* < .05), we conducted multivariable MR (MVMR) to validate the putative causal association (Figure 1).^37^ Multivariable MR simultaneously includes genetic instruments of all exposures in the same model, thus accounting for potential confounding of one exposure by the other (eg, potential confounding of the association between WMH and AD by SBP). Finally, for exposures with significant MVMR association, the following sensitivity analyses were conducted: (1) Qhet-MVMR to account for confounding due to weak instruments^38^ and (2) bidirectional MR to confirm the causal direction (eMethods in Supplement 2). Causal estimates are scaled to represent a 1-SD change for continuous exposures and per 1-unit higher log odds for binary exposures. Analyses were performed using R, version 3.3.2 (R Project for Statistical Computing) and the TwoSampleMR, CAUSE-MR, and MVMR R packages. We used matSPDlite^39^ to correct for multiple testing^40^; based on the correlation matrix between exposures, we identified 3 independent phenotypes leading to a *P* value threshold of *P* < .02 (.05/3).

### Statistical Analysis

#### Analyses on Individual-Level Data

Statistical analysis was performed from July 26, 2020, through July 24, 2022. We conducted individual-level data analyses in longitudinal prospective cohort studies to examine the association of genetically determined WMH burden, stroke, and BP traits with incident dementia, while addressing potential selective survival bias.^41^

##### Primary Analyses

Analyses were conducted in 13 longitudinal cohorts participating in the CHARGE (Cohorts for Heart and Aging Research in Genomic Epidemiology) consortium with cognitive assessment periods ranging from 1981 to 2016^42^ and large biobanks (Trøndelag Health Study, Estonian Biobank, and UK Biobank) assessed between 1987 and 2018. Nearly all cohorts were population based, except MEMENTO (memory clinic patients without dementia and with cognitive symptoms), with an assessment period from 1979 to 2014. Dementia diagnosis was based on standard criteria (eMethods in Supplement 2).

We used Cox proportional hazards regression models to examine the association of genetic risk scores for WMH, stroke, and BP traits with incident all-cause dementia. For each exposure, we constructed wGRS based on the weighted sum of alleles of independent genome-wide significant risk variants for the corresponding exposure (the same variants as for genetic instruments in 2SMR analyses), using effect estimates from the GWAS that they were derived from as weights.^43^ The wGRS were standardized (mean of 0, variance of 1), so that each unit change in the wGRS corresponds to 1-SD increase. Analyses were restricted to participants with no dementia at baseline and at least 1 follow-up visit. The Cox proportional hazards regression model used age as the time scale and was adjusted for sex, principal components of population stratification, and educational level (a strong determinant of cognitive function, associated with socioeconomic status and vascular risk factors; eTable 3 in Supplement 1). Data were censored at the age at dementia diagnosis or last follow-up. Cohort-specific estimates were combined using a fixed-effects inverse variance–weighted meta-analysis. Sensitivity analyses were conducted to rule out confounding by stroke, given the established association of WMH burden with stroke risk and of stroke with risk of dementia^13^: we excluded individuals with a stroke history at inclusion and adjusted for interim stroke (ie, occurring between blood draw and dementia diagnosis or end of follow-up), except in the Charles F. and Joanne Knight Alzheimer Disease Research Center biobank. As in the 2SMR, *P* < .02 was considered significant, accounting for 3 independent exposures.^40^

##### Secondary Analyses

Additional analyses were conducted in 3C and AGES, 2 large longitudinal population-based cohort studies with participants aged 65 years or older (eMethods in Supplement 2). We first examined whether survival bias during follow-up might affect our results using illness-death models,^44^ accounting for interval censoring of time to onset of dementia and competing risk of death. Second, we examined associations of genetically determined vascular exposures (WMH, stroke, BP) with incident dementia subtypes (all-cause dementia, AD, vascular and/or mixed dementia; eMethods in Supplement 2) at more liberal instrument selection thresholds (*P* value between .50 and 5 × 10^−8^) using polygenic scores (PGSs). A value of *P* < .02 correcting for 3 independent traits was considered statistically significant.

## Results

### Characteristics of Study Populations

For 2SMR analyses, the GWASs used to derive genetic instruments comprised up to 757 601 individuals of European ancestry: WMH GWASs included 48 454 individuals (mean [SD] age, 66.0 [7.5] years; 57.6% women); stroke GWASs included 67 162 cases and 454 450 controls (mean [SD] age, 63.7 [8.4] years; 44.8% women); and BP GWASs included 757 601 individuals (mean [SD] age, 56.8 [8.0] years; 54.2% women). The GWASs used for the dementia outcome comprised 75 024 cases and 397 844 controls for AD-meta and 21 982 cases and 41 944 controls for clinically diagnosed AD (mean [SD] age at AD onset, 75.5 [4.4] years; 56.9% women).^13,15,24,25,26^

For individual-level analyses, the 13 longitudinal cohorts included 157 698 participants of European ancestry, of whom 10 699 developed incident all-cause dementia (mean [SD] age at baseline, 64.2 [11.3] years; mean [SD] age at dementia diagnosis, 74.4 [9.1] years; 55.4% women; follow-up ranged from 3 to 25 years). The AGES and 3C studies used for secondary analyses comprised 978 and 621 incident dementia cases, respectively; a mean (SD) age at baseline of 75.9 (5.3) years and 74.1 (5.4) years, respectively; a mean (SD) age at dementia diagnosis of 85.1 (4.7) years and 81.8 (5.4) years, respectively; and a follow-up of 10.2 and 7.7 years, respectively.

### Associations of WMH, Stroke, and BP With AD Risk Using Summary-Level Data

**Figure 2.**
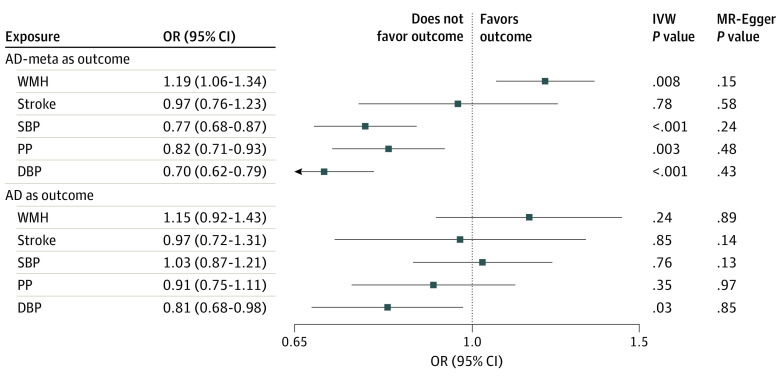
Mendelian Randomization Results of Vascular Risk Factors With Alzheimer Disease (AD) Point estimates and 95% CIs from the inverse variance weighted (IVW) method, along with the *P* value for the IVW and MR-Egger intercept, are shown. The causal estimates are scaled to represent a 1-SD change for the continuous exposures and per 1-unit higher log odds for binary exposures. DBP indicates diastolic blood pressure; OR, odds ratio; PP, pulse pressure; SBP, systolic blood pressure; and WMH, white matter hyperintensity.

**Figure 3.**
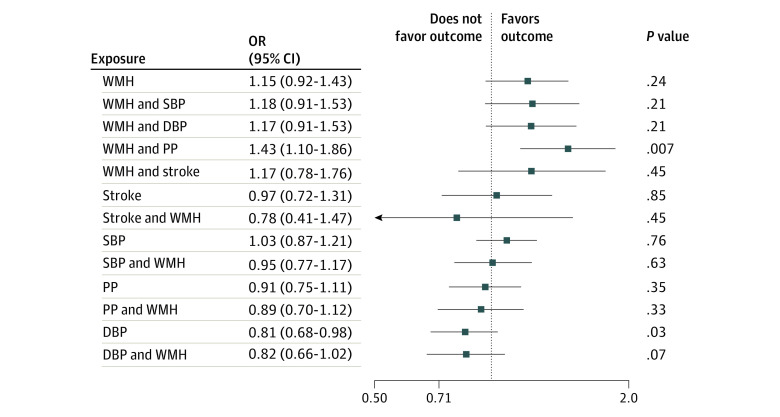
Multivariable Mendelian Randomization (MVMR) Along With the Univariable Mendelian Randomization (MR) for Alzheimer Disease (AD) as the Outcome Univariable MR and MVMR results are shown, and the association *P* values are shown on the far right. The causal estimates are scaled to represent a 1-SD change for the continuous exposures and per 1-unit higher log odds for binary exposures. DBP indicates diastolic blood pressure; OR, odds ratio; PP, pulse pressure; SBP, systolic blood pressure; and WMH, white matter hyperintensity.

The genetic instruments for WMH, stroke, and BP were strongly associated with the exposures (*F* = 22-65; eTables 1 and 2 in Supplement 1). Using the inverse variance weighting method, we found significant associations of genetically determined larger WMH burden (odds ratio [OR], 1.19 [95% CI, 1.06-1.34]; *P* = .008) and lower DBP (OR, 0.70 [95% CI, 0.62-0.79]; *P* < .001), SBP (OR, 0.77 [95% CI, 0.68-0.87]; *P* < .001), and PP (OR, 0.82 [95% CI, 0.71-0.93]; *P* = .003) with AD-meta risk and of lower DBP with clinically diagnosed AD risk (OR, 0.81 [95% CI, 0.68-0.98]; *P* = .03) (Figure 2; Table). The complementary MR tools MR-RAPS, weighted median and mode, robustly ruled out uncorrelated pleiotropy (eTables 4B and 5B in Supplement 1). The bayesian MR-CAUSE method that additionally accounts for correlated pleiotropy further supported a causal association of WMH with both AD and AD-meta, with a posterior distribution of the causal model distinctively different from the sharing model (ΔELPD = 0.91 for AD and 0.50 for AD-meta) (Table; eTable 6 in Supplement 1). On the contrary, stroke and BP traits suggested a better fit of the sharing model with potential unmeasured confounders for AD-meta (stroke, ΔELPD = –2.60; SPB, ΔELPD = –3.00; DBP, ΔELPD = –2.20) and AD (stroke, ΔELPD = 0.41; SPB, ΔELPD = 0.44; DBP, ΔELPD = –1.20) (Table). For associations of WMH with AD, although there was a better fit of the causal model (ΔELPD = 0.91), a significant proportion of genetic instruments appeared to be shared with unmeasured confounders (*P* < .001 for the sharing model) (Table; eFigure 1 in Supplement 2). We therefore performed a multivariable analysis, adjusting for the associations of closely related traits using MVMR. Greater genetically determined WMH burden was associated with a 43.4% increase in the probability of AD risk (OR, 1.43; 95% CI, 1.10-1.86; *P* = .007, per unit increase in WMH risk alleles) after accounting for PP associations (Figure 3; eTable 7 in Supplement 1), a 28.6% increase in disease risk compared with univariable estimates (OR, 1.15; 95% CI, 0.92-1.43; *P* = .24), with consistent direction of association. A bidirectional MR analysis between the WMH and PP suggested a causal path of higher PP with larger WMH burden (eTable 8 in Supplement 1).

**Table.** Suite of 2-Sample MR Analyses With AD Outcomes

Exposure	MR-IVW method		MR-CAUSE			MVMR	
	Odds ratio	*P* value	ΔELPD^a^	*P* value for causal effect	*P* value for shared model	OR	*P* value
**Vascular risk factors associated with AD-meta**							
WMH	1.19 (1.06 to 1.34)	.008	0.50	.39	.62	NA	NA
Stroke	0.97 (0.76 to 1.23)	.78	−2.60	.005	.27	NA	NA
SBP	0.77 (0.68 to 0.87)	<.001	−3.00	<.001	<.001	NA	NA
PP	0.82 (0.71 to 0.93)	.003	−1.60	<.001	.001	NA	NA
DBP	0.70 (0.62 to 0.79)	<.001	−2.20	<.001	<.001	NA	NA
**Vascular risk factors associated with clinically defined AD**							
WMH	1.15 (0.92 to 1.43)	.24	0.91	.77	<.001	1.43 (1.10 to 1.86)	.007
Stroke	0.97 (0.72 to 1.31)	.85	0.41	.42	.52	NA	NA
SBP	1.03 (0.87 to 1.21)	.76	0.44	NA	.16	NA	NA
PP	0.91 (0.75 to 1.11)	.35	−0.97	NA	.42	NA	NA
DBP	0.81 (0.68 to 0.98)	.03	−1.20	.05	.09	NA	NA

Abbreviations: AD, Alzheimer disease; DBP, diastolic blood pressure; IVW, inverse variance weighting; MR, mendelian randomization; MVMR, multivariable MR; NA, not applicable; PP, pulse pressure; SBP, systolic blood pressure; WMH, white matter hyperintensity; ΔELPD, change in expected log pointwise posterior density, testing causal vs sharing model.

^a^ ΔELPD > 0 indicates a better fit for the causal model.

### Association of WMH, Stroke, and BP wGRS With Incident Dementia Using Individual-Level Data

**Figure 4.**
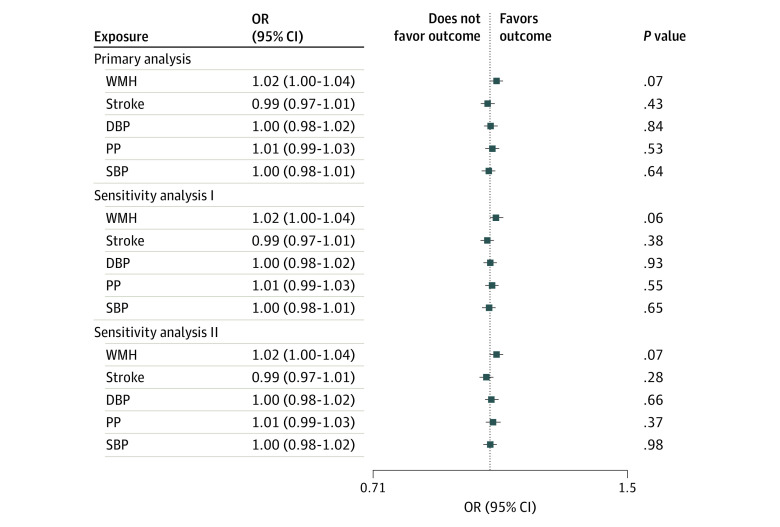
Meta-Analysis Results of Risk Factor–Weighted Genetic Risk Scores (per SD Increase) With Incident All-Cause Dementia Primary analysis: Cox proportional hazards regression model adjusted for sex, principal components of population stratification, study-specific criteria, and educational level. Sensitivity analysis I: Cox proportional hazards regression model adjusted for sex, principal components of population stratification, study-specific criteria. Sensitivity analysis II: prevalent stroke excluded and the Cox proportional hazards regression model adjusted for sex, principal components of population stratification, study-specific criteria, and interim stroke status. Association *P* values are shown on the far right. DBP indicates diastolic blood pressure; OR, odds ratio; PP, pulse pressure; SBP, systolic blood pressure; and WMH, white matter hyperintensity.

In a meta-analysis of 13 longitudinal cohort studies, we observed a nonsignificant association of larger genetically determined WMH burden with increased risk of incident all-cause dementia (hazard ratio [HR], 1.02; 95% CI, 1.00-1.04; *P* = .07, per SD increase in WMH wGRS) (Figure 4; eTable 9 in Supplement 1). After adjustment for educational level and interim stroke, this association remained substantially unchanged (Figure 4). There was no significant heterogeneity across cohorts (*I*^2^ = 7%; *P* = .38) (eFigure 2 in Supplement 2). Genetic liability to stroke and genetically determined BP traits failed to show significant associations with incident all-cause dementia, with negative point estimates for stroke and SBP. All exposures showed at least nominally significant associations with increased mortality, most significantly for SBP (HR, 1.04; 95% CI, 1.03-1.06; *P* = 1.9 × 10^−14^); the association of WMH with mortality was no longer significant after adjusting for educational level or interim stroke status (eTables 9 and 10 in Supplement 1).

In secondary analyses, using illness-death models for 2 older population-based cohorts (3C and AGES), genetically determined higher WMH burden, BP levels, and genetic liability to stroke were not associated with incident all-cause dementia, with effect estimates similar to those observed in Cox proportional hazards regression models (eTable 11 in Supplement 1), thus ruling out potential biases related to competing risk of death during follow-up in the context of interval censoring.

In further secondary analyses using PGSs, we found that PGSs for WMH and stroke with less stringent instrument-significance thresholds (eTable 12 in Supplement 1) were significantly associated with increased risk of all-cause dementia in both cohorts (eFigures 3 and 4 in Supplement 2 and eTables 13 and 14 in Supplement 1). In sensitivity analyses excluding prevalent stroke and adjusting for interim stroke, WMH PGS associations with dementia remained unchanged, while stroke PGS associations were markedly attenuated in both cohorts. Meta-analyses of effect estimates from 3C and AGES (for PGS bins at *P* < .50) showed significant associations of WMH and stroke PGS with increased risk of all-cause dementia, AD, and vascular or mixed dementia (eTable 15 in Supplement 1). Blood pressure PGSs were mostly not associated with dementia, except for protective associations of SBP and DBP PGSs with AD in AGES only, attenuated after excluding prevalent stroke and adjusting for interim stroke (eTable 16 in Supplement 1).

## Discussion

Using comprehensive 2SMR workflow–leveraging summary statistics of large GWASs for vascular traits (WMH, stroke, and BP) and AD, we report a putative causal association of genetically determined larger WMH burden with increased risk of AD, both clinically diagnosed^25^ and using parental history of dementia as a proxy.^26^ The former association was strengthened after accounting for PP using multivariable MR. Blood pressure traits showed evidence for a protective association with AD, with evidence for confounding by shared genetic instruments. In longitudinal individual-level analyses across 13 cohorts and biobanks with 157 698 participants, we observed a nonsignificant trend toward an association of larger WMH burden with incident all-cause dementia. Although all vascular exposures were associated with mortality, there was no evidence for selective survival bias during follow-up in secondary analyses using illness-death models in AGES and 3C. In these cohorts, PGSs for WMH and stroke were associated with all-cause dementia, AD, and vascular or mixed dementia, and for WMH, these associations were independent of interim stroke.

Overall, of all vascular phenotypes considered, WMH appeared to show the most robust associations with dementia risk, including AD, AD-meta, and all-cause dementia, adding evidence of causal associations to findings from observational studies^12,45,46,47,48^ and highlighting WMH as a key pathway to target for dementia prevention (eFigure 5 in Supplement 2). This finding reinforces earlier observations of a putative causal association of WMH with AD-meta,^13^ expanding it to a larger AD-meta GWAS^26^ and to clinically diagnosed AD.^25^ The stronger association of WMH with the latter after accounting for PP, with a marked (28.6%) increase in AD risk, is intriguing. Pulse pressure is a marker of arterial stiffness,^49,50^ which was shown to be associated with WMH burden and amyloid-β deposition and its progression in the brain.^51,52^ Elevated PP may dysregulate brain endothelial cells and increase cellular production of oxidative and inflammatory molecules, possibly leading to amyloid-β secretion and blood-brain barrier breakdown.^53,54,55^

High BP is the strongest known risk factor for WMH, with MR studies suggesting a causal association, even among persons without clinically defined hypertension.^13^ Moreover, BP-lowering treatments were shown to slow WMH progression in randomized trials,^56,57,58,59,60^ especially with intensive BP lowering.^60^ Given the aforementioned associations of WMH with AD, the association of high BP with lower risk of AD and AD-meta in the 2SMR analysis appears counterintuitive. However, it aligns with earlier MR studies using instruments from smaller BP GWASs or genetic proxies for BP-lowering effect.^16,17,18,19,61^ Our sensitivity analyses using MR-CAUSE suggest that pleiotropic effects from unmeasured confounders might explain this unexpected directionality of association, highlighting the importance of such examinations rather than merely removing or downweighing pleiotropic variants (MR-RAPS, weighted median and mode). Moreover, while we did not observe selective survival bias during follow-up, given the late age of dementia onset (mean age, 85 years),^62^ the strong association of genetically determined high BP with premature death, in line with observational studies,^63,64,65,66^ raises the possibility of selective survival bias before study entry. The apparently protective effect of high BP on dementia risk might thus reflect underlying collider bias^67^ rather than causality.^68,69,70^ Although nonsignificant, the association of PP and DBP with incident all-cause dementia had point estimates above 1 in the longitudinal cohort studies, which are probably less exposed to selective survival than the AD case-control GWAS used for the 2SMR analyses.^25,26^ Beyond these possible biases, our results highlight the complexity of the epidemiologic association between BP and dementia risk, with strong age effects. High BP in midlife but not late life was shown to be associated with dementia risk,^71,72,73^ and in a meta-analysis of longitudinal cohorts, the reduction in AD risk associated with antihypertensive medication use was greater among younger compared with older participants with hypertension.^74^ Meta-analyses of clinical trials have shown the effectiveness of antihypertensive medication in reducing the combined outcome of dementia and cognitive impairment, while evidence for dementia alone remains inconclusive.^74,75^

In contrast to BP measurements, which show high intraindividual variability,^76^ WMH volume is a more stable marker, reflecting white matter damage secondary to changes in the structure and/or function of cerebral small vessels. Assuming that WMH at least partly mediates the association of BP with dementia in the population, WMH may better capture the brain damage caused by BP than BP itself. White matter hyperintensity likely also reflects the association of other parameters with white matter integrity, such as cerebral amyloid angiopathy or factors associated with the resilience of the brain white matter to vascular insults. Given the high prevalence of WMH in the general population among stroke-free individuals,^48^ our results highlight WMH as a major causal pathway to consider for the prevention of dementia.

### Limitations

This study has some limitations. First, despite the large samples used for 2SMR, we observed imprecise estimates for certain associations (stroke and BP traits). This finding could be attributed to comparatively weaker instruments (the stroke *F* statistic was lower than for other exposures)^77^ or to limitations of certain MR methods for exposures comprising very large numbers of genetic instruments (eg, BP traits).^78,79^ Second, the AD-meta phenotype that uses family history of dementia as a proxy for AD enables the increase in sample size and also possibly includes more patients with mixed dementia, who are likely underrepresented in GWASs using clinically defined AD only, although they represent most dementia cases in the population. However, the imprecision of the AD-meta phenotype is a limitation; therefore, we have provided additional analyses focusing exclusively on clinically defined AD. Third, single-exposure MR analyses might oversimplify underlying causal associations, and therefore complementary approaches investigating more broadly the dementia exposome are warranted.^80^ Fourth, in our longitudinal analyses, the number of incident dementia cases remained modest, with some differences in ascertainment methods, which may have limited power to detect associations. Although secondary exploratory analyses showed an association of PGS for genetically determined WMH with incident dementia subtypes, these require validation in independent datasets, especially as our multiple testing correction did not account for the dementia subtypes analyzed. Fifth, validation of our findings in populations of non-European ancestry, as larger datasets become available, will be crucial.

## Conclusions

Our findings provide converging evidence that WMH is a major vascular factor associated with dementia risk, emphasizing that it should be prioritized in preventive efforts. They also support WMH as a surrogate marker for clinical trials to prevent dementia by controlling vascular risk.^60,81^ Our results prompt caution when interpreting MR studies with late-onset diseases, particularly when survival is strongly associated with the exposure instruments, and highlight the importance of combining complementary analytical approaches and applying them to several independent studies to mitigate study-specific limitations and biases.

## Notes

Supplement 1. **eTable 1.** Instrument Strength of the Risk Factors Determined Using F-Statistics (Univariable MR) **eTable 2.** Conditional F-Statistic (FTS) in Multivariable MR **eTable 3.** Cohort Characteristics **eTable 4a.** Two-Sample MR (2SMR) of Vascular Risk Factors With ADmeta **eTable 4b.** Pleiotropy Robust 2SMR Results of Risk Factors With ADmeta **eTable 5a.** Two-Sample MR (2SMR) of Vascular Risk Factors With AD **eTable 5b.** Pleiotropy Robust 2SMR Results of Risk Factors With AD **eTable 6.** CAUSE Testing the Sharing vs Causal Model for ADmeta and AD as the Outcomes **eTable 7.** Multivariable Mendelian Randomization Results of Risk Factors With AD **eTable 8.** Bidirectional Relationship Between WMH and PP **eTable 9.** wGRS Association of Exposures With Incident All-Cause Dementia and Mortality **eTable 10.** wGRS Association of Exposures With Incident All-Cause Dementia and Mortality: Sensitivity Analyses **eTable 11.** Illness Death Model (IDM, i.e., Multistate Model) Association Results for the Different Risk Factors With All-Cause Dementia **eTable 12.** Instrument Strength of the Risk Factors per Polygenic Score Bins Determined Using F-statistics **eTable 13.** WMH Polygenic Profile Score (PGS) Association With Dementia Outcomes in 3C and AGES **eTable 14.** Stroke Polygenic Profile Score (PGS) Association With Dementia Outcomes in 3C and AGES **eTable 15.** Meta Analysis of PGS Association for WMH and Stroke With Dementia Outcomes in 3C and AGES **eTable 16a.** SBP Polygenic Profile Score (PGS) Association With Dementia Outcomes in 3C and AGES **eTable 16b.** DBP Polygenic Profile Score (PGS) Association With Dementia Outcomes in 3C and AGES **eTable 16c.** PP Polygenic Profile Score (PGS) Association With Dementia Outcomes in 3C and AGES

Supplement 2.
eMethods.
**eFigure 1.** Comparison of the Sharing and the Causal Model Using CAUSE for AD as the Outcome **eFigure 2.** Forest Plot Showing Association of WMH-wGRS With Incident All-cause Dementia (Study-Wise) **eFigure 3.** WMH Polygenic Profile Score (PGS) Association With Dementia Outcomes in 3C (Blue) and AGES (Black) **eFigure 4.** Stroke Polygenic Profile Score (PGS) Association With Dementia Outcomes in 3C (Blue) and AGES (Black) **eFigure 5.** Central Role of WMH With Dementia Outcomes
eReferences.

Supplement 3.
Data Sharing Statement
