# Supplementary material for: Genetic Complexities of Cerebral Small Vessel Disease, Blood Pressure, and Dementia
Source: JAMA Netw Open. 2024 May 22;7(5):e2412824. doi: 10.1001/jamanetworkopen.2024.12824 (PMC11112447; doi:10.1001/jamanetworkopen.2024.12824)
Supplement: Supplement 3. — Data Sharing Statement [file jamanetwopen-e2412824-s003.pdf]

Data Sharing Statement

Sargurupremraj. Genetic Complexities of Cerebral Small Vessel Disease, Blood Pressure, and Dementia. *JAMA Netw Open*. Published May 22, 2024.
doi:10.1001/jamanetworkopen.2024.12824

Data
Data available: No

Additional Information
Explanation for why data not available: The study includes publicly available data and individual-level longitudinal data from multiple cohorts and biobanks that can be leveraged through collaboration upon contacting the respective study coordinators. Please refer to the study-specific information provided in the supplementary methods.
